# Impact of an Immune Modulator Mycobacterium-w on Adaptive Natural Killer Cells and Protection Against COVID-19

**DOI:** 10.3389/fimmu.2022.887230

**Published:** 2022-05-04

**Authors:** Sarita Rani Jaiswal, Jaganath Arunachalam, Ashraf Saifullah, Rohit Lakhchaura, Dhanir Tailor, Anupama Mehta, Gitali Bhagawati, Hemamalini Aiyer, Bakulesh Khamar, Sanjay V. Malhotra, Suparno Chakrabarti

**Affiliations:** ^1^ Cellular Therapy and Immunology, Manashi Chakrabarti Foundation, New Delhi, India; ^2^ Department of Blood and Marrow Transplantation, Dharamshila Narayana Super-Speciality Hospital, New Delhi, India; ^3^ Amity Institute of Molecular Medicine and Stem Cell Research, Amity University, Noida, India; ^4^ Department of Cell, Development & Cancer Biology and Center for Experimental Therapeutics, Knight Cancer Institute, Oregon Health & Science University, Portland, OR, United States; ^5^ Department of Pathology and Microbiology, Dharamshila Narayana Super-speciality Hospital, New Delhi, India; ^6^ Research & Development, Cadila Pharmaceuticals Ltd, Ahmedabad, India

**Keywords:** Mw for COVID-19 *Mycobacterium w* (Mw), COVID-19, SARS-CoV-2, innate immunity, NKG2C, adaptive NK cells, NKG2A, ADCC

## Abstract

The kinetics of NKG2C^+^ adaptive natural killer (ANK) cells and NKG2A^+^inhibitory NK (iNK) cells with respect to the incidence of SARS-CoV-2 infection were studied for 6 months in a cohort of healthcare workers following the administration of the heat-killed *Mycobacterium w* (Mw group) in comparison to a control group. In both groups, corona virus disease 2019 (COVID-19) correlated with lower NKG2C^+^ANK cells at baseline. There was a significant upregulation of NKG2C expression and IFN-γ release in the Mw group (p=0.0009), particularly in those with a lower baseline NKG2C expression, along with the downregulation of iNK cells (p<0.0001). This translated to a significant reduction in the incidence and severity of COVID-19 in the Mw group (incidence risk ratio-0.15, p=0.0004). RNA-seq analysis at 6 months showed an upregulation of the ANK pathway genes and an enhanced ANK-mediated antibody-dependent cellular cytotoxicity (ADCC) signature. Thus, Mw was observed to have a salutary impact on the ANK cell profile and a long-term upregulation of ANK-ADCC pathways, which could have provided protection against COVID-19 in a non-immune high-risk population.

## Introduction

The rapid explosion of the novel coronavirus, SARS-CoV-2, in early 2020, across the globe overwhelmed even the most prepared health infrastructures (1) and exposed the healthcare workers to an unforeseen situation, where they remained at the greatest risk of exposure to the highest viral load, in the absence of prevention or cure. Despite a very high incidence of infections, witnessed in the Indian population as well, there was a surprising sparing of the urban slum-dwellers (2). We hypothesized the role of a bolstered innate immune system secondary to chronic pathogen exposure as a plausible reason for this phenomenon. We reasoned that due to a ubiquitous exposure to cytomegalovirus (CMV) in early childhood, followed by the exposure to a multitude of pathogens subsequently, there might be a stronger repertoire of NKG2C expressing adaptive natural killer (ANK) cells in this population (3).

Early CD56^bright^ NK cells have a very high expression of NKG2A, which functions as an inhibitory checkpoint in the process of functional maturation of NK cells (4). Both NKG2C and NKG2A bind to the same ligand, Human Leukocyte Antigen-E (HLA-E), but the latter binds with severalfold greater affinity compared to NKG2C (5). Unlike somatic mutations witnessed in adaptive immune cells to produce precise and clonal antigen specificity, NK cells express a plethora of germline-encoded activating and inhibitory receptors. The regulation of NK cell function, which is termed as “licensing,” occurs with a stochastic expression of killer-immunoglobulin-like receptors (KIRs), which bind to self-HLA-class 1 antigens with biallelic specificity (6). The expression of KIRs for which appropriate self-HLA antigens exist enables a continued inhibition of NK cells preventing autologous cytotoxicity. Hence, NKG2A^+^inhibitory natural killer (iNK) cells are key to the prevention of the autoreactivity of NK cells, prior to the KIR-driven process of licensing. In a subset of mature and licensed NK cells, exposure to CMV leads to the expression of a C-lectin type activating receptor, NKG2C, which is encoded by the *KLRC2* gene (7). These cells are characterized by the upregulation of NKG2C and the downregulation of the inhibitory counterpart, NKG2A (8). This subset of NK cells, now called NKG2C^+^ANK cells, exhibits the classic adaptive features, such as clonal expansion, persistence, and recall memory more akin to memory cytotoxic T cells than canonical NK cells (9).

While the major subset of ANK cells expresses NKG2C, which is the defining phenotype, several myeloid (FCER1G, PLZF) and B lymphoid (SYK, EAT-2) genes are downregulated, and certain T lymphoid genes (CD3ζ, BCL11B) are upregulated in ANK cells (10). The alterations in these gene expressions in ANK cells favor an augmentation of antibody-dependent cellular cytotoxicity (ADCC). In a small subset of ANK cells, the adaptive functions might be demonstrable with epigenetic distribution of myeloid and lymphoid associated genes as described above, even without the upregulation of NKG2C expression (10, 11). Hence, for the sake of clarity, the ANK cells described in this study are NKG2C^+^ ANK cells.

In the context of haploidentical hematopoietic cell transplantation (HCT), NKG2C^+^ANK cells were found to afford protection, not only against leukemia, but also a range of viral infections, other than CMV. It was suggested that high NKG2C^+^ ANK cells were essential in maintaining a non-inflammatory milieu without compromising antiumor effect post-HCT (12–15). Along with these seminal findings, the existing evidence suggest that certain natural infections, such as Hantavirus, as well as vaccinations for influenza and bacille Calmette-Guerin (BCG) are capable of upregulating NKG2C^+^ANK cells in CMV-exposed populations (16–18). We hypothesized that a novel heat killed *Mycobacterium w* (Mw), also known as *Mycobacterium indicus pranii*, an approved immunomodulator in India (19), which has been used in the treatment of severe COVID-19 (20), might upregulate NKG2C^+^ANK cells and offer protection against SARS-CoV-2 (severe acute respiratory syndrome coronavirus-2) infections in the process. We studied the impact of Mw on the incidence of COVID-19 (corona virus disease 2019 caused by SARS-CoV-2) in a cohort–control study in front-line healthcare workers and its impact on the kinetics and repertoire of NKG2C^+^ANK cells in the context of the KLRC2 genotype.

## Materials and Methods

In a single-center non-randomized cohort control study, 50 Healthcare Workers (HCWs) from a single department in the hospital were administered 0.1 ml *Mw* (Sepsivac; Cadila Pharmaceuticals, Ahmedabad, India) intradermally in each arm on day 1 of the study (Mw group) and 50 randomly selected HCWs from the rest of the institution were enrolled in a “control group”. In addition, in the Mw group, those without any local site reaction, who consented for second dose, were administered an additional dose of 0.1 ml Mw, 30 days after the first dose. The observation period of the study was time-censored from 15 days after administration of the first dose of Mw to 180 days (September 2020 to February 2021), based on the available evidence for evaluating a clinical or immunological response to vaccines against SARS-CoV-2 (21).

HCWs from both groups were involved in regular care of suspected or proven COVID-19 patients with complete personal protective equipment (PPE). All HCWs included in the study had nasopharyngeal swab evaluated for SARS-CoV-2 by reverse transcriptase–polymerase chain reaction (RT-PCR), on development of symptoms suggestive of COVID-19 or following unprotected contact with an individual with COVID-19, either at home or hospital. The body temperature, pulse rate, oxygen saturation, and self-reporting of symptoms was evaluated before and after each working day (22). COVID-19 was diagnosed, and its severity was graded as per established criteria. The duration of observation was 6 months from September 2020 to February 2021. In addition, blood was collected, at baseline, days 30, 60, and 100 from all the subjects, for evaluating the kinetics of NKG2C^+^ANK cells and T cells. All the subjects of this study also underwent evaluation for CMV status (seropositive or seronegative).

All subjects provided written informed consent for participating in the study. The study was approved by the institutional ethics committee and registered with Clinical Trials Registry of India (CTRI/2020/10/028326).

### Real Time Reverse Transcription Polymerase Chain Reaction for Detection of SARS-CoV-2

All Covid tests were done by Truenat real-time RT-PCR test. Nasopharyngeal swabs were collected using a standard nylon-flocked swab and inserted into the viral transport medium provided from the same company (Molbio Diagnostics Pvt. Ltd., New Delhi, India). Samples were transported immediately to the laboratory, maintaining proper temperature, and processed as per manufacturer’s guidelines (Truenat Beta CoV Chip-based real-time PCR test for Beta Coronavirus; Molbio Diagnostics Pvt. Ltd., India). The target sequence for this assay is the *E* gene of Sarbeco virus and human RNaseP (internal positive control). Confirmatory genes were the *RdRP* gene and *ORF1A* gene.

### Immunological Monitoring

Peripheral blood mononuclear cells (PBMCs) were isolated from whole blood samples, by density gradient centrifugations using HiSep™ LSM 1077 media. For surface staining, 0.5 × 10^6^ cells were washed with phosphate-buffered saline and stained with the following antibodies that were used for phenotypic analysis: CD3(APC-H7, SK-7) CD16 (PE-Cy7, B73.1), CD56 (APC R700, NCAM16.2), CD57 (BV605, NK-1), NKG2A (PE-Cy7, Z199), CD4 (APC-H7), CD8 (Per-CP Cy), CD45RA (FITC), and CD45RO (BV605), from BD Biosciences, (San Jose, CA, United States) and NKG2C (PE, REA205) from Miltenyi Biotec (Bergisch Gladbach, Germany). Cells were then incubated for 30 min. Viability was assessed with 7-AAD viability dye (Beckman Coulter). For intracellular staining, cells were stained for IFN- γ using monoclonal antibodies for interferon-gamma (IFN-γ) (4S.B3) and perforin (Alexa647, DG9) (BD Biosciences) after fixation and permeabilization with appropriate buffer (BD Biosciences and e-Biosciences, San Diego, CA, United States). Flow cytometry was performed using 10 color flow cytometry (BD FACS Lyrics), and the flow cytometry data were analyzed using FlowJo software (v10.6.2, FlowJo). Unstained, single-stained (one antibody/sample), and fluorescence-minus-one samples were used as controls for the acquisition as well as the subsequent analysis. Statistical divergences were determined by the GraphPad Prism software.

The gating strategy has been described earlier (15). NKG2C^+^ANK cells were defined as the CD56^dim^NKG2C^+^NKG2A^-^CD57^+^ subset of NK cells. The NKG2C/NKG2A ratio was calculated as the relative percentage of NKG2C^+^NKG2A^−^ ANK cells/NKG2C^-^NKG2A^+^ iNK cells (**Figure S1**).

### Interferon-Gamma Assay

This was carried out as described earlier (15). In brief, fresh PBMCs from patients were isolated as above and resuspended in the Roswell Park Memorial Institute Medium (RPMI) media with 10% fetal bovine serum (GIBCO) along with penicillin and streptomycin (5,000 U/ml; Thermo Fisher Scientific, Waltham, Ma, United States). They were incubated with IL-15 (10 ng/ml) [IL-15 recombinant human protein; Thermo Fisher Scientific, CA, United States) for 6 h. The IL-15 dose was standardized based on IFN-γ expression studies on NK cells from normal donors, where IFN-γ expression was limited to 1%-5% with 95% confidence interval (data not shown). Brefeldin A and monensin protein transport inhibitors (BD Biosciences) were added 2 h into the assay. The cells were then stained with mononuclear antibodies against cell surface CD3, CD56, NKG2D, NKG2A, and NKG2C followed by fixation permeabilization and intracytoplasmic monoclonal antibodies for IFN-γ (4S.B3, BD Biosciences).

### KLRC2 (NKG2C) Genotyping

The *KLRC2* gene encodes for NKG2C and is located in chromosome 12p13. For categorizing subjects KLRC2 wild-type homozygous (*Wt^+^/Wt* ^+^), KLRC2 deletion homozygous *(Del^+^/Del^+^)*, and KLRC2 heterozygous (*Wt^+^/Del^+^*), PCR-based KLRC2 genotyping was carried out. DNA was isolated from the peripheral blood using QIAGEN QIAamp@ DNA blood mini kit method (Hilden, Germany). PCR amplification was carried out with forward and reverse primer sequences as previously described (23). PCR amplification was carried out in 20 μl volume, containing 1x PCR master mix, which has premixed taq polymerase, dNTPs, PCR buffer (Thermo Fisher Scientific, Waltham, MA 02451, United States), 1.65 pmol forward primers and reverse primers for KLRC2 deletion and wild-type *KLRC2* genes (see **Table S1**), 100 pg-1 μg genomic DNA. Amplification was performed using a T100 thermal cycler (Bio-Rad, Hercules, CA, United States). Cycling temperature profiles were adopted from a previous study (23) with minor modifications.

Briefly, the reaction mixture was subjected to one cycle of denaturation at 95°C for 10 min followed immediately by 29 cycles of 95°C for 20 s, 50°C for 30 s, 72°C for 40 s; and a final extension at 72°C for 10 min before cooling to 4°C. A non-template control was included in each batch of PCR reactions. PCR products were identified by running the entire PCR product on a 2% agarose gel for 60 m at 70 V. The size of the amplicons was determined by comparison against the migration of a 100-bp DNA ladder (GeneDireX, Inc, Taoyuan County, Taiwan). Agarose gels were visualized and documented using the Gel doc XR+ gel documentation system (Bio-Rad, Hercules, CA, United States). An illustration of the PCR assay is shown in **Supplementary Figure** (**Figure S2**).

### RNA-seq Analysis

This was carried out on both Mw and Control group at 6 months following exposure to Mw. Four subjects from each group without COVID-19 were selected for the study.

### RNA Isolation Using Trizol Method and polyA RNA Selection

Peripheral blood mononuclear cells (PBMCs) were isolated from whole blood samples by density gradient centrifugations using HiSep™ LSM 1077 media (Himedia, Mumbai, India). Approximately 5 × 10^6^ PBMCs were used for RNA isolation using the Trizol method and followed by DNase treatment for RNA purification. Purified RNA was quantified using a Qubit 4.0 fluorometer. Approximately 5 μg of total RNA was used for the polyA RNA selection using NEB NEXT oligo d(T)_25_ beads (NEB, MA, USA). PolyA RNA was quantified and integrity assessed using a Qubit 4.0 fluorometer (Invitrogen, Waltham, MA, USA) according to manufacturer instructions.

### Complementary DNA Library Preparation and Whole Transcriptome Sequencing Using MinION 2.0-Oxford Nanopore Technologies

For direct cDNA native barcoding sequencing (SQK-DCS109 with EXP-NBD104; Oxford Nanopore Technologies, Oxford, United Kingdom), 100 ng of polyA RNA was used for the library preparation. Using H minus Reverse Transcriptase (Invitrogen, Waltham, MA, United States), complementary DNA (cDNA) was synthesized, followed by RNA degradation and a second-strand synthesis of cDNA. Double-stranded cDNA was used for the end preparation, followed by native barcoding and adaptor ligation (all steps were followed, according to the manufacturer’s instructions). Ligated cDNA was loaded on the flow cell (R9) in MinION. Libraries were sequenced, specifying 72 h on the Oxford Nanopore Technologies (ONT) MinION using R9.4.1 flow cells and MinKNOW (v21.06.10, Microsoft Windows OS based) to generate FAST5 files. FAST5 files were base-called with Central Processing Unit (CPU)-based Guppy basecaller (v.5.0.11) (ONT) to create summary text files and FASTQ files of the reads for further downstream analysis.

### Sample Pre-Processing and Quality Assessment

The demultiplexing of the pooled samples and adapter removal was carried out using inbuilt algorithm of Minknow. Linux Long Time Support (v.20.04) Operating System was used for all the analysis. A comprehensive report of the sequencing was generated by NanoComp (https://github.com/NanoComp/h5utils) tool, and a sequencing quality assessment was done by the FastQc tool (https://www.bioinformatics.babraham.ac.uk/projects/fastqc/). Sample reads were subjected to a minimum phred quality score of 9 and reads with lower quality were filtered out using NanoFilt (https://github.com/wdecoster/nanofilt) tool. Some initial reads are usually prone to low base-calling quality. Hence, 50 bp of each initial reads from every sample were filtered out for quality maintenance using the NanoFilt tool only. All the samples were subjected to quality assessment before and after quality filtering.

### Differential Gene Expression Analysis

Differential gene expression analysis (DGE) was done using “pipeline-transcriptome-de” (https://github.com/nanoporetech/pipeline-transcriptome-de) pipeline. This pipeline from nanopore tech uses snakemake, minimap2, salmon, edgeR, DEXSeq, and stageR to automate DGE workflows on long read data. The pipeline was set to make only reads aligned to minimum 3 samples to be considered for analysis. A separate conda (https://docs.conda.io/en/latest/#) environment was created on Linux OS to host this pipeline. Quantification and DGE was done by the R language-based (https://www.r-project.org/) tool edgeR (https://bioconductor.org/packages/release/bioc/html/edgeR.html) employing gene-wise negative binomial regression model and normalization factor (transcript mean of M-value) for each sequence library. Differentially expressed genes (DEGs) with log2Fold change (log2Fc) ≥0.5, ≤-0.5 and associated p-value <0.05 were selected as significant for further analysis. The annotation of DEGs was fetched from the ENSEMBL database (https://asia.ensembl.org/index.html) using R language biomaRt package (https://bioconductor.org/packages/release/bioc/html/biomaRt.html). All the file compilations were ultimately done using Microsoft Excel and Libre Office calc.

Hierarchical clustering analyses was performed between all four samples of each group to generate a heatmap from normalized log2 counts per million expression values using web-based START (Shiny Transcriptome Analysis Resource Tool, https://kcvi.shinyapps.io/START/) tool. A comparative gene expression boxplot on the basis of log2 counts per million between the Mw and control group was generated from the START tool. A volcano plot involving all DEGs were created using R language-based ggplot2 (https://ggplot2.tidyverse.org/) package.

### Gene Ontology Pathway Analysis

Pathway enrichment analysis was done for significant DEGs using g:Profiler (https://biit.cs.ut.ee/gprofiler/gost) using Gene Ontology. The pathways related to ANK, ADCC, and innate immune inflammatory pathways were considered for focused analysis. Functional enrichment was done by R-based clusterProfiler (v. 4.2.1) https://bioconductor.org/packages/release/bioc/html/clusterProfiler.html tool. An adjusted p-value threshold of ≤0.05 was considered for this study.

### Statistics

Lymphocyte subsets have been represented as % of the parent population. Binary variables were compared between the groups using a chi-square test. The continuous variables were analyzed using an independent sample t-test considering Levene’s test for the equality of variances and non-parametric tests (Mann–Whitney U test). A P-value <0.05 was considered to be significant. GraphPad Prism (version 8.0 for Windows; GraphPad Software, La Jolla, CA, United States) was used for the statistical assessment (unpaired low-parametric Mann–Whitney or Kruskal–Wallis test and Spearman correlation). Recursive partitioning analysis was carried out using the *rpart* package (https://cran.r-project.org/web/packages/rpart/index.html) in R (https://cran.r-project.org/) to generate optimal cut-off for ANK cells at baseline.

The efficacy of Mw in reducing the incidence of COVID-19 was calculated in terms of attack rates incidence risk ratio (IRR), absolute risk reduction (ARR), and intervention efficacy (see **Table S2**). This was calculated in terms of infections occurring at 2 weeks after the administration of Mw is customary in prophylactic studies as well as those occurring any time between during the study period.

## Results

### Mw and COVID-19

The characteristics and outcomes of the Mw and control groups are detailed in **Table 1**.

**Table 1:** Characteristics and outcomes.

	Control group (N=50)	Mw group (N=50)	p-value
Age at vaccination, median (range), Years	28 (21-55)	28 (22-56)	0.11
<45	45	42	
>45	5	8	
Gender			0.3
Male	34	28	
Female	16	22	
SARS-CoV-2 infection	17	3* (6)^#^	0.0012
Mild	11	3* (3)^#^	0.02
Moderate	6	0	0.01
Severe	0	0	1.0
Incidence rate/10,000 person days	24.05* (18.8)^#^	3.57* (6.6)^#^	
Time to infection, median (range), days	56 (18-135)	151 (2-159)	<0.0001

*Time-censored observation period (days 15-180).

^#^Uncensored observation period (days 0-180).

All subjects were seropositive for CMV. The median exposure to COVID-19 patients without PPE was 2 (range, 0-4) for the Mw group and 1 (range, 0-3) for the control group (p=0.9).

**Figure 1:**
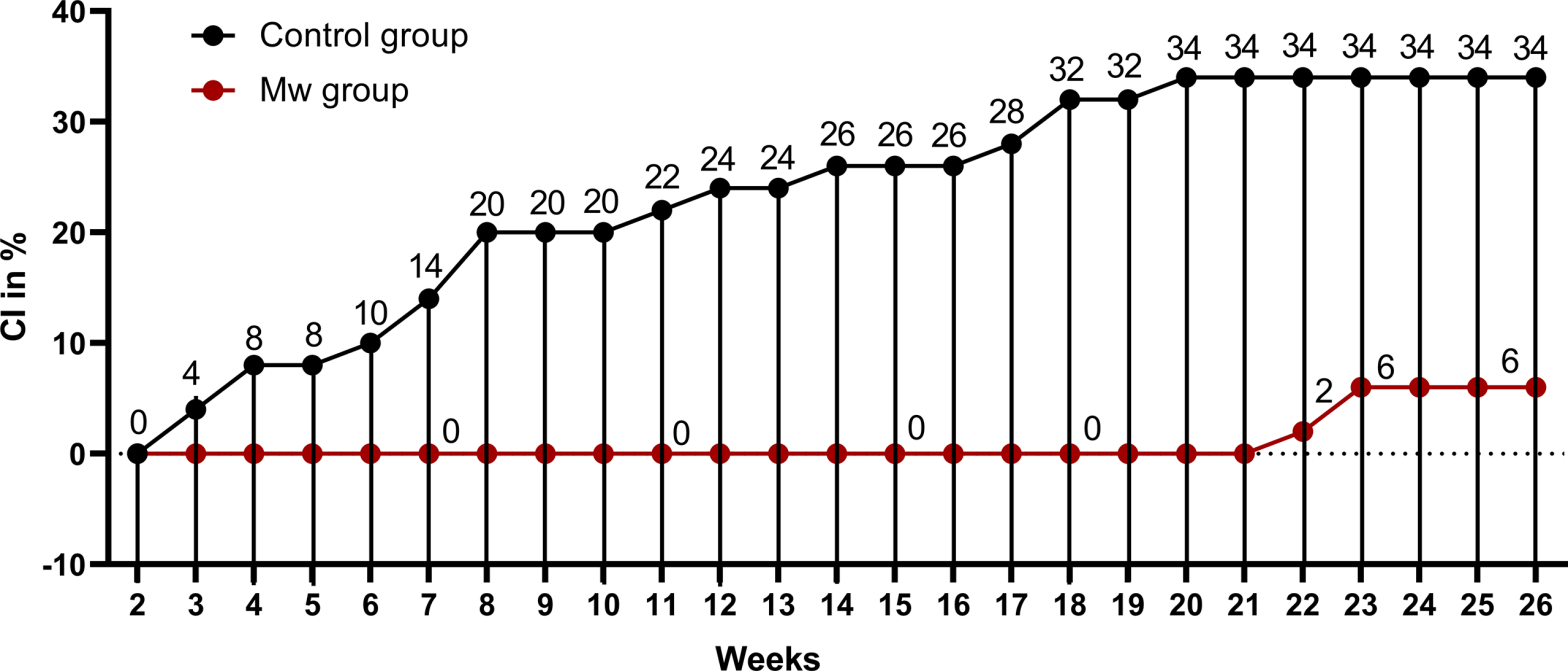
Impact of Mw vaccination on COVID-19 compared to a control group: Points and connecting line plot show the infection trend in Mw group (n=50) and the control group (n=50). The *x*-axis shows the time in weeks and *y*-axis shows the cumulative incidence (CI) in %. Red- and black-shaded circles represent Mw group and control group, respectively.

During the planned observation period of the study (15 days after administration of the first dose of Mw to 180 days), only 3 infections were observed in the Mw group vs. 17 in the control group (p=0.0008) (**Figure 1**), with an ARR of 0.28. The time-censored incidence rates (IRs) of COVID-19 of 3.57 and 24.05/10,000 person-days in Mw and control groups, respectively (IRR-0.15, 95% CI, 0.04-0.5, p=0.0004), efficacy rate for Mw of 82.35%, (95% CI, 50%-96%) and an HR of 0.15 (95% CI, 0.04-0.5, p=0.003) (**Table 2**).

**Table 2:** Incidence rate ratio of SARS-CoV-2 infections and Mw efficacy.

Parameter	Estimate	*p*-value	95% CI
Incidence rate ratio (IRR)	0.15* (0.35)^#^	0.0004* (0.02)^#^	0.04 to 0.5*
			(0.15 to 0.81)^#^
Attack rate in Mw control (ARU)	0.34* (0.34)^#^		
Attack rate in Mw treated (ARV)	0.06* (0.12^)#^		
Mw efficacy (%)	82.35%*		50%–96%*
	(64.7%)^#^		(19%–84%)^#^
Absolute risk reduction (ARR)	0.28* (0.22)^#^		
Number needed to treat (NNT)	3.6* (4.54)^#^		

*Time-censored observation period (day 15-180).

^#^Uncensored observation period (day 0-180).

However, during the uncensored observation period beginning immediately after the administration of Mw (0-180 days), 23 subjects developed COVID-19, 6 in the Mw group vs. 17 in the control group (p=0.02), with the absolute risk reduction (ARR) of 0.22, the IRs of COVID-19 of 6.66 and 18.7/10,000 person-days in Mw and control groups, respectively (IRR-0.35, 95% CI, 0.15-0.81, p=0.02), the efficacy rate for Mw of 64.7%, (95% CI, 19%-84%) and a hazard ratio (HR) of 0.31(95% CI, 0.12-0.78, p=0.01). All infections on the Mw group were mild, while 6 of 17 in the control group had moderate disease and required hospital admission (p=0.01). No deaths were recorded in either group. Out of 6 infections in the Mw group, 3 infections each occurred before 15 days and beyond 150 days, whereas 17 subjects had COVID-19 at a median of 56 days (range, 18–135) in the control group.

Twenty-four subjects in the Mw group were randomized 1:1 to receive the second dose, 30 days after the first dose. The impact of the second dose on protection against COVID-19 could not be analyzed as none of the randomized subjects developed COVID-19 during the study period.

### Safety Profile of Mw

Mw was safe with no systemic adverse effects. Only one subject had mild fever (37.5°C) for less than 2 h, 24 h after administration, which was self-limiting. However, 14% had pain at the local sites, with 12% developing pain and erythema lasting for more than 72 h. In 8% of the subjects, ulceration was noted at the local site with long-term scars similar to that observed with BCG vaccination. Those with severe local reactions were all older (35 years and above), compared to the median age of the group (28 years).

### Higher Baseline NKG2C^+^ANK Cells Correlated With a Lower Risk of COVID-19

Baseline data on immune parameters were available in 80 subjects in the overall cohort, 30 in the control and 50 in the Mw group. The The NKG2C^+^ANK cells in those who got infected (n=16) were 7.9 ± 6.1%, (including 3 who got infected within 7 days of receiving Mw) compared to 20.9 ± 13.8% in those who remained uninfected (p= 0.0005).

**Figure 2:**
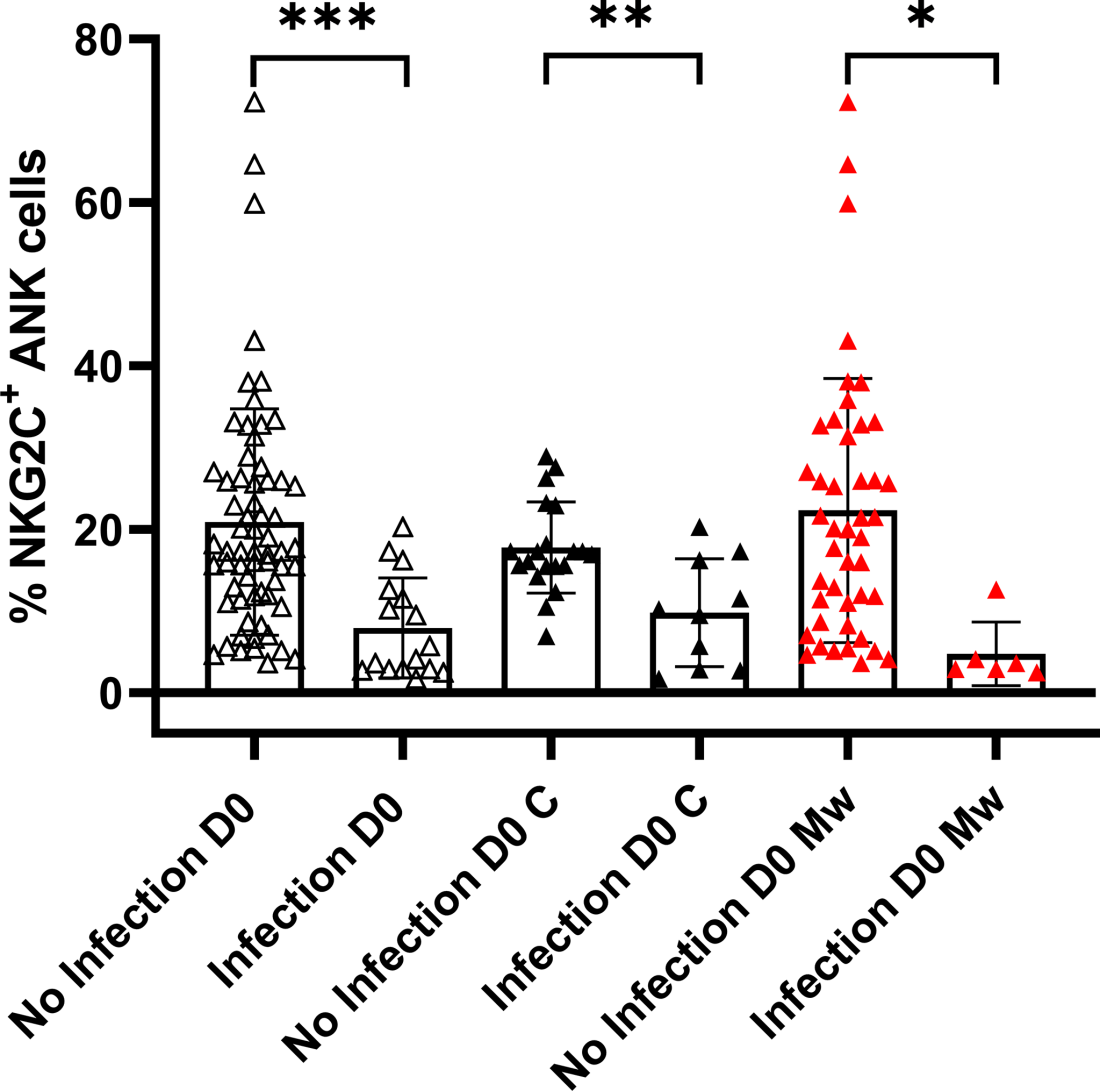
Relationship between NKG2C^+^ANK cells and COVID-19: Scatter dot with bar plot showing expression of NKG2C^+^ ANK cells at baseline in the overall cohort (no infection, n=64 and infection, n=16), control group (no infection, n=20 and infection, n=10) and Mw group (no infection, n=44 and infection, n=6) with respect to SARS-CoV-2 infection. Black and red upside shaded triangles represent NKG2C^+^ ANK for Control group and Mw group respectively and unshaded upside triangles represent NKG2C^+^ ANK of both groups combined. ***p < 0.001, **p < 0.01 and *P < 0.05.

Among the 30 subjects in the control group, who were tested at baseline for NKG2C^+^ANK cells, 10 developed COVID-19. The NKG2C^+^ANK cells at baseline were 9.7 ± 6.6% in those with infection compared to 17.7 ± 5.56% in those without infection (p=0.0016). All 6 subjects who acquired the infection in the Mw group, also had a lower baseline NKG2C^+^ANK (4.76 ± 3.9% vs 22.3 ± 16.1%, **Figure 2**, p=0.01). Interestingly, all 3 subjects experiencing COVID-19 in the Mw group beyond 150 days, had low baseline ANK cells and 2 of the 3 subjects did not show any increment in NKG2C^+^ANK levels or NKG2C/NKG2A ratios at any of the time-points. We did not find any correlation between expression of NKG2A as well as NKG2C/NKG2A ratio with development of symptomatic COVID-19 in this study.

A cut-off of 15% was established for NKG2C^+^ANK cells based on recursive partitioning for evaluating the effect of NKG2C upregulation. Among the evaluable cohort of 80 subjects, only 3 out of 46 subjects in the “high ANK” group (>15%) had COVID-19, compared to 13 out of 34 in the “low ANK” group (<15%), with an ARR of 0.32 and an IRR of 0.17 (95% CI, 0.05-0.54, p=0.0006) and HR of 0.14 (95% CI, 0.1-0.48, p=0.002). The protective efficacy of “high ANK” levels (>15%) was 84% (95% CI, 45-94.6). The protective effect of “high ANK” was evident in both Mw (p=0.006) and control (p=0.01) groups, when analyzed separately. Even as a continuous variable, baseline ANK maintained its inverse correlation with COVID-19 (HR-0.84, 95%CI-0.7-0.9, p=0.0001)

### Mw and ANK Cells

Thirty subjects from the Mw group and 15 from the control group, who did not develop COVID-19 or any other significant infection during the study period, were included in the final longitudinal analysis of immunological impact of Mw.

### Baseline ANK Levels Did Not Differ, but ANK Kinetics at 60 Days Were Different in the Mw Group

**Figure 3:**
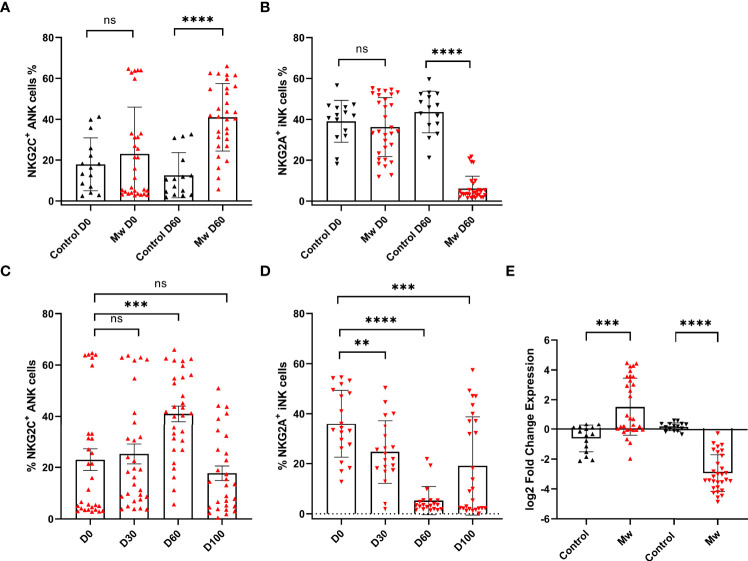
Impact of Mw on both ANK cells and iNK) cells: **(A)** Scatter dot with bar plot showing the expression of NKG2C^+^ ANK cells (Mw group -n=30 and control group- n=15) and **(B)** NKG2A^+^ iNK cells (Mw group-n=30 and control group- n=15) with and without of Mw vaccine at baseline and day 60. **(C)** Scatter dot with bar plot showing kinetics of NKG2C^+^ ANK cells, and **(D)** NKG2A^+^ iNK cells expression in Mw group (n=30) at baseline, day 30, day 60, and day 100. **(E)** log2FC expression of NKG2C^+^ and NKG2A^+^ in both Mw group (n=30) and vontrol group (n=15) at day 60 after normalization with baseline. Red upside and downside shaded triangles represent NKG2C^+^ ANK and NKG2A^+^ iNK, respectively, for Mw group. Black upside and downside shaded triangles represent NKG2C^+^ ANK and NKG2A^+^ iNK, respectively, for control group. ****p < 0.0001, **p <0.01, ***p <0.001, and ns, p-value not significant.

There was no difference in overall NK cells between the two groups at baseline or at subsequent time-points. NKG2C^+^ANK and NKG2A^+^ iNK cell expressions were also similar at baseline in both groups (**Figures 3A, B**). The data from the control group shown in further comparisons only pertain to the baseline and day 60, as the values in the control group for both NKG2C^+^ ANK cells and NKG2A^+^ iNK cells did not change significantly at any of the time-points.

In the Mw group, on the other hand (**Figures 3A–D**), NKG2C^+^ANK cells increased from baseline (23.0 ± 22.9%) to peak at day 60 (40.9 ± 16.5%, day 60 p=0.0009, **Figure 3C**), while NKG2A^+^ iNK cells reduced from 36.23 ± 14.4% to 26.22 ± 14.04% on day 30 and 6.17 ± 6.01% on day 60 (p<0.0001, **Figure 3D**). The absolute values as well as the log two-fold change (log2FC) were significantly different for both NKG2C^+^ANK cells (1.5 ± 1.9% vs -0.62 ± 0.89%, p=0.0002, **Figure 3E**) as well as NKG2A^+^ iNK cells (-2.9 ± 1.2% vs 0.17 ± 0.29%, p<0.0001, **Figure 3E**) cells at day 60, compared to the control group.

### The Impact of Mw on NKG2C Upregulation Was Only Observed in Those With Low ANK Cells at Baseline, but Not for NKG2A^+^ iNK Downregulation

**Figure 4:**
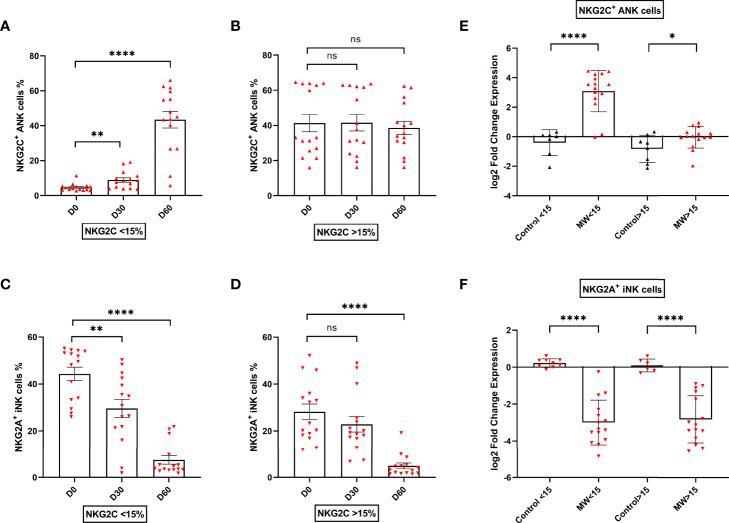
Impact of Mw on upregulation of NKG2C^+^ ANK cells with respect to NKG2C expression at baseline: Scatter dot with bar plot showing the kinetics of **(A)** NKG2C^+^ ANK cells (n=15) and **(C)** NKG2A^+^ iNK (n=15) cells expression in Mw group at baseline, days 30 and 60 in respect to <15% NKG2C at baseline. **(B)** NKG2C^+^ ANK (n=15) cells and **(D)** NKG2A^+^ iNK (n=15) cell expression in Mw group at baseline, days 30 and 60 in respect to >15% NKG2C at baseline. Log2FC expression of **(E)** NKG2C^+^ ANK and **(F)** NKG2A^+^ iNK cells at day 60 after normalization with baseline value in Mw group (>/<15%, n=15) and control group. Red upside and downside shaded triangles represent NKG2C^+^ ANK and NKG2A^+^ iNK respectively for Mw group. Black upside and downside shaded triangles represent NKG2C^+^ ANK and NKG2A^+^ iNK, respectively, for control group. ****p < 0.0001, **p < 0.01, *p < 0.05, and ns, p-value not significant.

The upregulation of NKG2C^+^ ANK cells was seen in those with NKG2C^+^ ANK levels below 15% (low ANK group). From a baseline value of 4.7 ± 2.2%, this increased to 8.9 ± 4.9 on day 30 (p=0.005) and 43.3 ± 19.12% on day 60 (p<0.0001, **Figure 4A**). This was also reflected in the log2FC values.

In those with NKG2C^+^ANK levels above 15% (kigh NKG2C^+^ANK), there was no significant change in the absolute values of NKG2C^+^ANK cells at either day 30 or day 60(**Figure 4B**), but a positive impact on log2FC was noted (p=0.04, **Figure 4E**).

The downregulation of NKG2A^+^iNK cells was noted in the Mw group, irrespective of baseline NKG2C expression (**Figure 4C, D**). The downregulation of NKG2A^+^iNK cells was significant at day 60 in the Mw group, irrespective of baseline NKG2C expression (-2.99 ± 1.21% vs. 0.23 ± 0.23%, p<0.0001 for low ANK group and -2.84 ± 1.28% vs 0.09 ± 0.35%, p<0.0001 for high ANK group) (**Figure 4F**).

**Figure 5:**
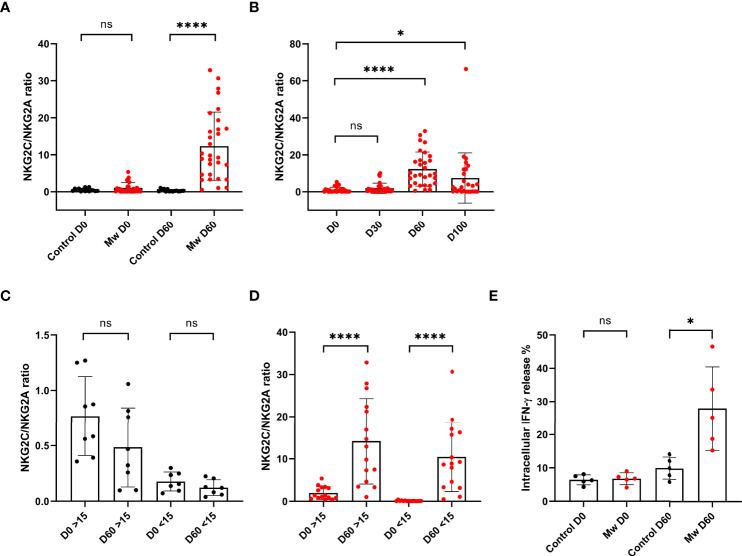
Mw showed sustained effect on NKG2C/NKG2A ratios until 100 days and Impact of Mw intracellular cytokine (IFN-γ) release: Scatter dot with bar plot showing **(A)** NKG2C/NKG2A ratio of control group (n=15) and Mw group (n=30) at baseline and day 60. **(B)** Kinetics of NKG2C/NKG2A ratio at baseline, days 30, 60, and 100 in Mw group. **(C, D)** Kinetics of NKG2C/NKG2A ratio at baseline and day 60 on the basis of >/< 15% NKG2C at baseline in Mw group and control group, respectively. **(E)** intracellular IFN-γ release in both control (n=5) and Mw (n=5) groups at baseline and day 60. Red and black shaded circles represent Mw group and control group, respectively. ****p < 0.0001, *p < 0.05 and ns, p-value not significant.

The NKG2C^+^ ANK/NKG2A^+^ iNK ratio was similar between two groups at baseline. However, this increased in the Mw group at day 60 (12.3 ± 9.2%, p<0.0001) and persisted until day 100 (7.44 ± 13.55%, p=0.01). This was witnessed for both low and high ANK group in the Mw cohort but not in the control cohort (**Figure 5A–D**).

### IFN-γ Release Was Higher in Mw Group at 60 Days

The IFN-γ release potential of the NKG2C^+^ANK cells was studied at baseline and at 60 days. This was similar between the groups at the baseline (mean-6.7 vs. 6.4). This was further studied among the same subjects at day 60. Cytokine release was significantly increased in the Mw group at day 60, compared to the control group (mean- 27.96 vs. 9.9%, p=0.01, **Figure 5E**). All subjects studied for cytokine release in the Mw group had shown a dominant ANK response. Unfortunately, the study lacked a comparison within the Mw group in terms of the ANK response and cytokine release.

### KLRC2 and Kinetics of ANK Cells

KLRC2 deletion was studied in the Mw group only. KLRC2 *Wt ^+^/del^+^* was detected in 36% of the subjects in the Mw group. This was not associated with any significant decrease in the baseline NKG2C^+^ANK levels (15.1 ± 17.67% vs. 20.4 ± 17.96%, p=0.46). There was no difference in the effect of Mw between the KLRC2 *Wt^+^/Wt* ^+^ and W*t^+^/Del^+^* groups at day 60 either (p=0.26), although the log2FC increase tended to be higher in the W*t^+^/Del^+^* group (p=0.12) (**Figures S3A, B**). KLRC2 deletion was detected in 2 out of 6 with COVID-19, compared to 16 out of 44 without COVID-19 (p=0.6).

### Mw Did Not Influence Kinetics of Naïve and Memory T Cell Subsets

CD4 and CD8 cells remained unchanged, as were the CD45RA and CD45RO subsets at days 30 and 60 in the Mw group (**Figures S4A, B**). The same was witnessed in the control group (data not shown).

NKG2A expression on CD3^+^ T cells was examined at baseline and subsequent timelines. Despite a significant downregulation of NKG2A observed in NK cells, no such effect was evident in the T cells (**Figure S4C**).

### Impact of the Second Dose of Mw on NKG2C^+^ANK Cells

**Figure 6:**
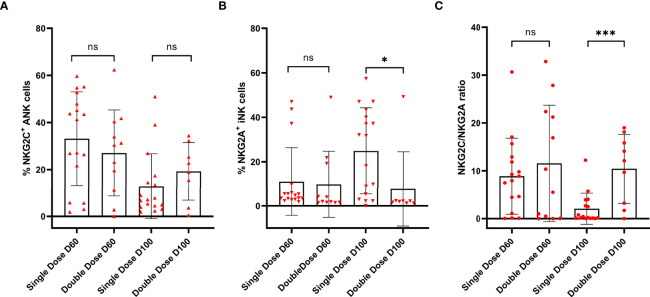
Impact of the second dose of Mw on ANK and INK cells: Scatter dot with bar plot showing expression of **(A)** NKG2C^+^ ANK (day 60, single dose; n=17, double dose; n=12 and day 100, single dose; n=17, double dose; n=12), **(B)** NKG2A^+^ iNK cells (day 60, single dose; n=12, double dose; n=12 and day 100, single dose; n=17, double dose; n=12) and **(C)** NKG2C/NKG2A (day 60, single dose; n=17, double dose; n=12 and day 100, single dose; n=17, double dose; n=12) ratio with respect to single and double dose of Mw vaccine at day 60 and 100. Red upside and downside shaded triangles represent NKG2C^+^ ANK and NKG2A^+^ iNK, respectively, for Mw group. Red-shaded circle represents Mw group. ***p < 0.001, *p < 0.05 and ns, p value not significant.

There was no difference between in NKG2C^+^ANK cells at day 60 and day 100 (p=0.28) in double vs. single doses of Mw (**Figure 6A**). However, the NKG2A^+^ iNK cell expression was significantly reduced at day 100 (7.7 ± 16.77% vs. 24.85 ± 19.43%, p=0.04) in the double-dose group (**Figure 6B**). The NKG2C/NKG2A ratio was also significantly higher in the double- dose group at day 100 (10.42 ± 7.19% vs. 2.08 ± 3.26%, p <0.001) (**Figure 6C**). There was no difference in CD4^+^ and CD8^+^ T cells and memory or naïve subsets at days 60 and 100 between the randomized groups (data not shown).

### RNAseq Analysis at 6 Months

Sequencing and mapping metrics along with quality scores are shown in **Supplement 1** (**Table S3**)

### Identification of Differentially Expressed Genes

Based on criteria of p-value < 0.05, a total of 3,603 out of 16,058 were found to be DEGs between the Mw and control groups, including 1,568 genes that were upregulated and 2,035 genes that were downregulated in the Mw group. Those DEGs with a log2 fold change of at least 0.5 were considered for further analysis. DEGs related to ANK cells and ADCC were selected out to analyze the differential expression pattern between two groups.

### ANK and ADCC Genes Were Upregulated in Mw Group

**Figure 7:**
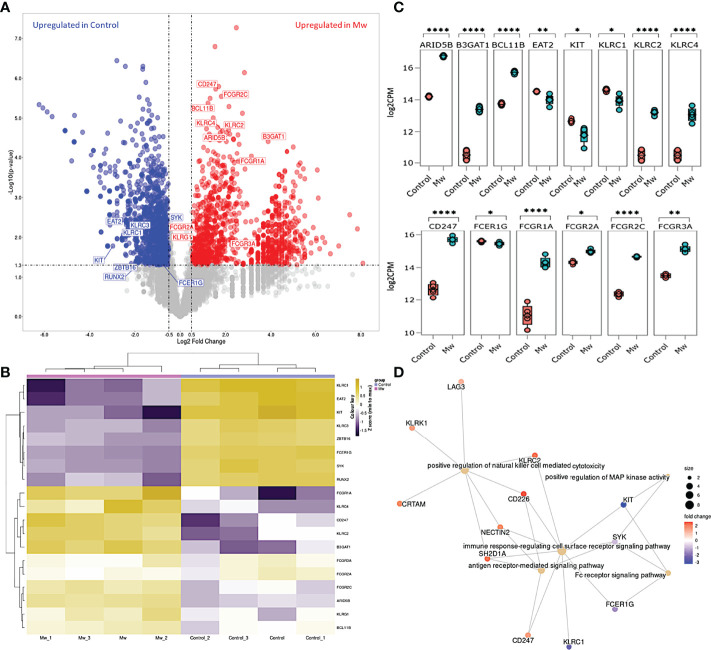
Differential gene expression and network analysis in Mw and Control group: **(A)** Volcano plot showing genes differentially expressed in Mw as compared to control group. Genes with a p-value <0.05 and log2 fold change ≥0.5 ≤-0.5 were considered significant. Selected genes are highlighted (Red: upregulated in Mw group, Blue: upregulated in control group). **(B)** Heatmap of RNA-seq expression data showing selected DEGs related to this study. Hierarchical cluster analysis was performed between all samples of Mw and Control groups. Gene expression is shown in normalized log2 counts per million. **(C)** Boxplots showing expression levels of 19 selected genes in Mw (green) as compared to control group (orange). Relative gene expression is shown in normalized log2 counts per million (log2CPM) for I) ANK pathway in the top panel, II) ADCC pathway in the middle panel. (*p-value < 0.05, **p-value < 0.01, ****p-value<0.0001). **(D)** GO network analysis of the top 5 enriched GO terms in the DEGs for adaptive NK cell and antibody-dependent cellular cytotoxicity (Fisher’s exact test using enrichGO function in R package clusterProfiler, multiple test correction by the Benjamini–Hochberg method, adj. p-value <0.05).

Nineteen DEGs were associated with ANK and ADCC pathways (**Figure 7A, B**). A total of 11 genes were upregulated and 8 were found to be downregulated in the Mw group. *KLRC2* (*NKG2C*), *BCL11B*, *ARID5B*, *B3GAT1* (*CD57*), and *KLRC4* were upregulated and *KLRC1*(*NKG2A*), *ZBTB16* (*PLZF1*), *KIT*, and *SH2D1B* (*EAT-2*) were downregulated in relation to the ANK pathway (**Figure 7C**). In the ADCC pathway, *CD247* (*CD3ζ*), *FCGR1A* (*CD64*), *FCGR2A*(*CD32a*), and *FCGR2C* (*CD32c*) were upregulated and *FCER1G* was downregulated (**Figure 7C**), favoring ANK-mediated ADCC.

### Gene Ontology Pathway Analysis

The pathway enrichment of DEGs were carried out on the selected genes focusing on ANK and ADCC pathways. The selected 19 DEGs were analyzed using goProfiler and clusterProfiler for Gene Ontology (GO). The GO analysis of the selected DEGs demonstrated that the immune response-regulating cell surface receptor signaling pathway (GO:0002768) and the positive regulation of NK cell-mediated toxicity (GO:0045954) were significantly upregulated (**Figure 7D**).

## Discussion

The identification of NK cells with the ability to persist and mount recall responses has challenged the traditional compartmentalization of adaptive and immune responses (24, 25). The NKG2C^+^ANK cells explored in this study are known to be induced by primary exposure to CMV (26, 27). However, in CMV-exposed patients, these NKG2C^+^ANK cells have been found to proliferate in response to a variety of viral pathogens, including HIV, Hepatitis C Virus (HCV), Hantavirus, influenza, and pox viruses (16, 28–31). The unique nature of this non-antigen-specific recall response against a wide variety of pathogens puts NKG2C^+^ANK cells at the forefront in the battle against novel pathogens such as SARS-CoV-2 (3, 32–36), where the antigen-specific adaptive response generated from T and B cells are absent at the outset of the pandemic.

The BCG vaccine has been shown to induce a long-term heterologous memory response of NK cells (33). This has been studied, both in the context of infections and cancer (37). In fact, when an epidemiological analysis of BCG was carried out with respect to its effect on COVID-19, a 10% increase in BCG uptake was associated with a similar decrease in mortality due to COVID-19 (38). Influenza vaccination has been shown to induce the proliferation of NKG2C^+^ANK cells with a potential for enhanced cytokine release (39). These considerations prompted us to explore this novel immunomodulator, Mw, which has been extensively studied as an adjuvant or prophylaxis in other infections (19, 40, 41), and some forms of cancers including BCG-resistant bladder cancer (42). In fact, a randomized study on the effect of Mw on the outcome of critically ill COVID-19 patients demonstrated early clinical benefit at 2–3 weeks (20). An ongoing randomized study is further evaluating the role of Mw in hospitalized non-critical patients with COVID-19 (NCT04358809).

Our study demonstrated that prophylaxis with Mw was associated with a sixfold reduction in the incidence of symptomatic COVID-19 in this high-risk cohort over a 6-month period. This protective efficacy was accompanied by a sharp increase in NKG2C^+^ANK cells between 30 and 60 days following exposure to Mw. A critical observation was a steep decline in the expression of the inhibitory counterpart, NKG2A^+^iNK cells, which continued through 100 days. This resulted in an increasing NKG2C/NKG2A ratio through the study period. Exposure to a second dose of Mw resulted in a continued downregulation of NKG2A^+^ iNK cell and a further increase in NKG2C/NKG2A ratios. The importance of the relative proportion of NKG2C- and NKG2A-expressing NK cells could be appreciated better in the context of the fact that the inhibitory impact of NKG2A overrides that of NKG2C, as it binds to the ligand HLA-E with severalfold greater affinity than NKG2C (5). In the context of SARS-CoV-2 infection, *in vitro* studies have demonstrated that the viral spike protein-1 (SP-1) upregulates NKG2A on NK cells and HLA-E on the infected lung epithelial cells, causing a strong inhibition of cytotoxicity of NK cells (43). Our group has demonstrated a correlation between the high expression of NKG2A, the suppression of NKG2C, and the adverse outcome following severe COVID-19 lung disease, despite viral clearance (36). It is possible that the adverse effect of KLRC2 deletion genotype as reported earlier (32), was mitigated by Mw. Hence, it might be inferred that if any intervention can achieve the reverse, i.e., downregulation of NKG2A and upregulation of NKG2C expression, this might offer innate protection against COVID-19.

In our study, lower baseline NKG2C^+^ANK cells were predisposed to COVID-19 in the control group. The same in the Mw group was associated with COVID-19 in the first 2 weeks but not thereafter until 150 days. It is also worth noting that the COVID-19 occurring in the Mw group beyond 150 days was associated with failure in the modulation of the adverse NKG2C^+^ANK cell profile in 2 out of 3 subjects. However, as the detection of antibodies against SARS-CoV-2 at the inclusion or the conclusion was not included as a part of this study, asymptomatic infections could have been missed. Thus, the effect of Mw interpreted from this study should be considered only with respect to symptomatic COVID-19.

Apart from phenotypic alteration, Mw seemed to influence the cytokine release potential as well, as evidenced by an increase in IFN-γ release at day 60 in the Mw group, compared to both baseline and the control group. Furthermore, the upregulation of NKG2C was dominantly noted in those with a lower baseline NKG2C^+^ANK cell. The upregulation of NKG2C expression might be more tightly controlled, unlike the downregulation of NKG2A, in mature and licensed NK cells, where an inhibitory regulation mediated by NKG2A is physiologically redundant due to KIR-mediated inhibitory control. More importantly, this also demonstrates that the downregulation of checkpoint receptor NKG2A is an important determinant of a favorable ANK profile. However, why certain individuals with KLRC2 *Wt^+^/Wt* ^+^ genotype but low ANK levels failed to alter the ANK profile despite exposure to Mw remains unexplained. This might be related to defects at the level of transcription or possibly translation and/or degradation of the protein, warranting further exploration.

In addition, as all our subjects were CMV seropositive, the impact of Mw on ANK kinetics in CMV-naïve individuals remains unexplored. Prior reports on the augmentation of the ANK population following vaccines or infections have been limited to CMV-seropositive subjects only (16, 39). Thus, it is possible that the effect of Mw might be muted in a CMV-unexposed population, as the epigenetic changes driving the transformation of a conventional NK cell to NKG2C+ANK cell are driven by CMV exposure (10).

**Figure 8:**
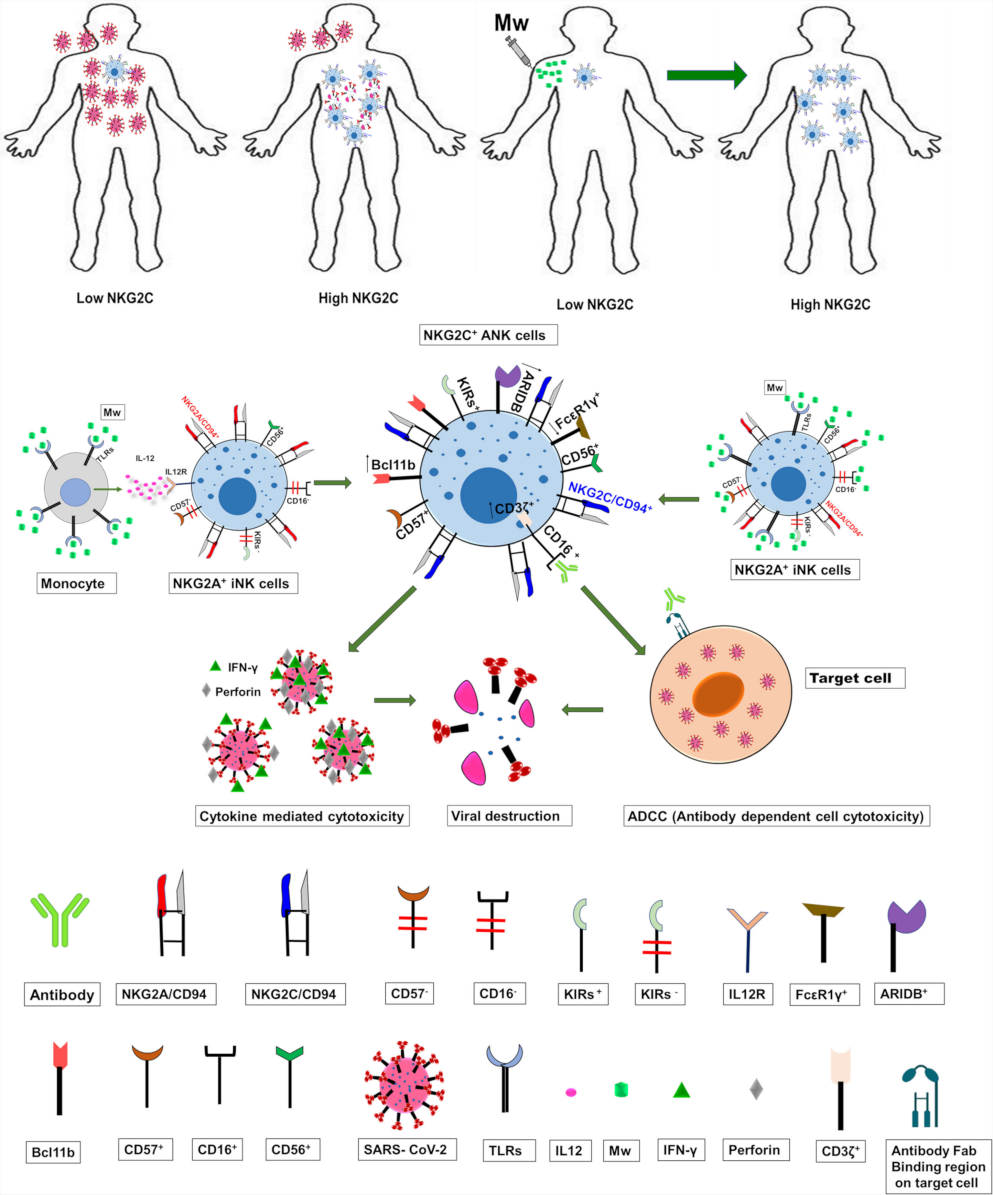
The suggested mechanistic pathway of the impact of Mw on ANK cells and protection against COVID-19.

Even though the phenotypic downregulation of NKG2C expression was noted at day 100 following Mw exposure, RNA-seq analysis suggested that the upregulation of the ANK pathway was evident at 6 months in the Mw group. Apart from the upregulation of KLRC2 and B3GAT1 and the downregulation of KLRC1, the key transcription factor in the ANK pathway, BCL11b, was persistently upregulated (44). The downregulation of EAT-2 and PLZF further corroborated the classic gene expression signature of ANK cells. Moreover, increased expression of AT-rich interaction domain 5B (ARID5B), as demonstrated in the Mw group, plays an important role in enhanced metabolism in ANK cells as well as increased IFN-γ release and survival (45). DGE analysis also revealed an enhancement of the ANK-mediated ADCC pathway, with significant upregulation of CD247 along with downregulation of FCER1G, which is a typical signature of ANK-ADCC (46). Both CD247 and FCER1G are adapter molecules for FCGRIIIA (CD16) with CD247 possessing 3 ITAMs against one ITAM of FCER1G, increasing the ADCC several folds (47). It is possible that the Mw-induced augmentation of NK-ADCC might potentiate the efficacy of SARS-CoV2 vaccines as well (48). Certain subgroups respond poorly to vaccines against SARS-CoV2, primarily those who are immunosuppressed due to disease or its treatment (49, 50), rendering them prime candidates for the exploration of Mw, either alone or as an adjuvant to the vaccine. If indeed there is a salutary effect on increasing the efficacy of the vaccine, a booster dose of Mw could augment this further, given the rapid waning of the neutralizing antibodies induced by the existing vaccines (51). Prior studies on Mw have explored its effect on TLRs *via* the monocyte/macrophage pathway (52–54) and not on NK cells. The suggested mechanistic pathway as to how Mw might be favorably influencing ANK-mediated protection against COVID-19 is depicted in **Figure 8**.

In conclusion, our study shows that Mw offered protection against COVID-19 in a high-risk population at the peak of the pandemic in the absence of antigen-specific immunity. This could be through a sustained upregulation of the NKG2C^+^ANK cell and ADCC pathway, along with simultaneous downregulation of NKG2A^+^iNK pathways. If borne out in a randomized setting, these findings might usher a novel approach to bolster heterologous immunity in the current and future pandemics and identify the ANK pathway in the immunological vulnerability for developing COVID-19.

## Data Availability Statement

The datasets presented in this study can be found in online repositories. The name of the repository and accession number (s) can be found at: NCBI Sequence Read Archive; accession number GSE197976 (https://www.ncbi.nlm.nih.gov/geo/query/acc.cgi?acc=GSE197976).

## Author Contributions

SJ, BK, and SC designed the study. AM, RL, GB, and HM performed the study. SJ, AS, AM, GB, and HM collected the data. SJ, AS, JA, SM, DT and SC analyzed the data. SJ and SC wrote the manuscript. All the co-authors reviewed and approved the manuscript.

## Funding

This study was supported by grant from Indo-US Science and Technology Forum (IUSSTF/VN-COVID/049/2020).

## Conflict of Interest

BK is employed by Cadila Pharmaceuticals Ltd, and SM serves as a scientific advisor to the Cadila Pharmaceuticals Ltd.

The remaining authors declare that the research was conducted in the absence of any commercial or financial relationships that could be construed as a potential conflict of interest.

## Publisher’s Note

All claims expressed in this article are solely those of the authors and do not necessarily represent those of their affiliated organizations, or those of the publisher, the editors and the reviewers. Any product that may be evaluated in this article, or claim that may be made by its manufacturer, is not guaranteed or endorsed by the publisher.
