# Supplementary material for: Impact of an Immune Modulator Mycobacterium-w on Adaptive Natural Killer Cells and Protection Against COVID-19
Source: Front Immunol. 2022 May 4;13:887230. doi: 10.3389/fimmu.2022.887230 (PMC9115578; doi:10.3389/fimmu.2022.887230)
Supplement: Supplementary file 2 [file DataSheet_2.pdf]

Supplemental Tables:

Table S1:

S.No.	Primers	Sequences
1.	KLRC2 *Wt* Forward primer	*5' CAGTGTGGATCTTCAATG 3'*
2.	KLRC2 *Wt* Reverse primer	*5' TTTAGTAATTGTGTGCATCCTA 3'*
3.	KLRC2 *Del* Forward primer	*5' ACTCGGATTTCTATTTGATGC 3'*
4.	KLRC2 *Del* Reverse primer	*5'ACAAGTGATGTATAAGAAAAAG 3'*

Table S2:

Statistical Method for calculation of efficacy of Mw

	Infected	Non-infected	Total
Mw treated	a	b	n1
Mw control	c	d	n2

Attack rate in Mw control (ARU) = c/n2

Attack rate in Mw treated (ARV) = a/n1

Incidence Risk Ratio (IRR)/ Relative Risk = ARV/ARU

Absolute Risk Reduction (ARR) = ARU- ARV

Number needed to treat (NNT) =1/ARR

Vaccine Efficacy (%) = ((ARU-ARV)/ARU)*100

95% CI Vaccine efficacy (%) = 1- IRR CI

Table S3:

<u>RNAseq Analysis</u>

Sequencing and mapping metrics

Average base calling phred quality score (q) was found to be more than 11 for each sample (range 8-30). Read base quality ≥ 9 was considered for DGE analysis. N50 of read length for sequenced pooled samples was more than 1kb (range 1kb-1.4kb). Average read-length was more than 800bp for each sample (range 200bp-4.6kb). Numbers of reads were more than 1 million for each sample. Total number of passed (>7 q score) base sequenced for each sample was found to be more than 700 million with one sample going as much as 1 billion implying a coverage of 21X to 35X. More than 60% mapping coverage was found for each sample. (See, Table S3)

Sequencing and mapping metrics of samples (pass reads)

Sample ID	Mapping Percentage	Number of reads	Mean read length (nt)	Average base quality score (q)
NB04_1 (Control)	67.20	1,582,130	868.4	11.2
NB04_2 (Control)	69.30	1,611,117	801.9	11.3
NB05_1 (Control)	66.76	1,561,297	946.2	11.3
NB05_2 (Control)	70.87	1,818,543	809.6	11.4
NB01 (Mw)	63.57	1,270,259	932.7	11.2
NB02 (Mw)	66.42	1,443,542	850.1	11.2
NB03 (Mw)	70.24	2,106,378	871.8	11.1
NB06 (Mw)	72.12	2,150,894	1039.8	11.3
